# Supplementary material for: On-shelf circulation of warm water toward the Totten Ice Shelf in East Antarctica
Source: Nat Commun. 2023 Aug 17;14:4955. doi: 10.1038/s41467-023-39764-z (PMC10435550; doi:10.1038/s41467-023-39764-z)
Supplement: Supplementary file 1 — Supplementary Information [file 41467_2023_39764_MOESM1_ESM.pdf]

Supplementary Information for

"On-Shelf Circulation of Warm Water Toward Totten Ice Shelf in East Antarctica"

Hirano et al.

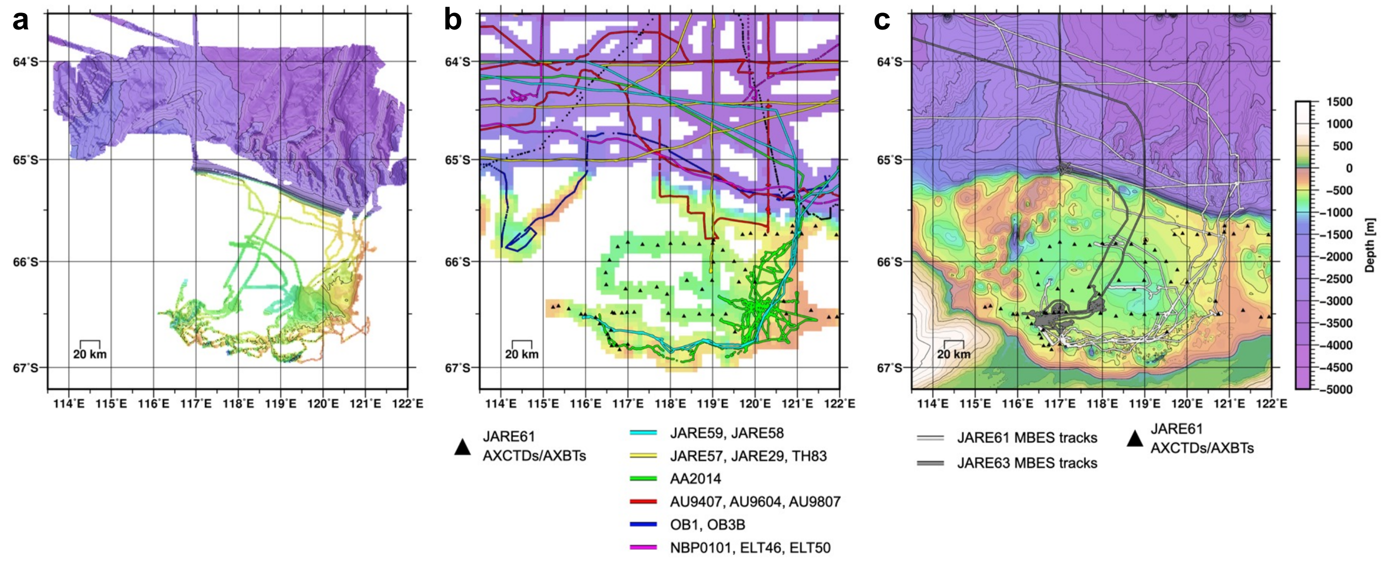

Supplementary Figure 1 | Bathymetric map off the Sabrina Coast. (a) Multibeam echo sounder (MBES) data, (b) representative depth data derived from single echo sounding data (lines and circles) and airborne expendable conductivity, temperature, and depth measurements (AXCTDs/AXBTs; triangles), and (c) compiled 1-km gridded digital terrain model utilized for physical oceanographic modeling. Ship tracks from the 61st and 63rd Japanese Antarctic Research Expedition (JARE61 and JARE63) are shown. AXCTD/AXBT locations from JARE61 are also displayed.

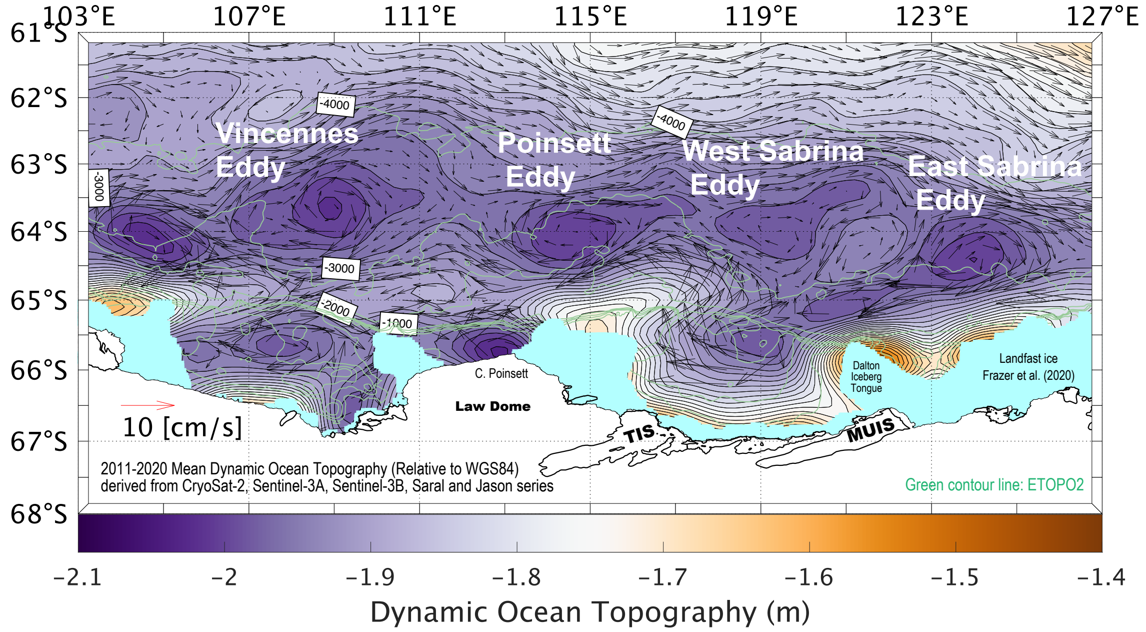

Supplementary Figure 2 | Multiple semi-permanent cyclonic eddies. Satellite-derived mean dynamic ocean topography[1] (DOT, m, color) and surface ocean current derived from DOT (cm s^{-1}, vectors) for 2011–2020. A cyclonic eddy train consists of the Vincennes Eddy, Poinsett Eddy, and West and East Sabrina Eddies.

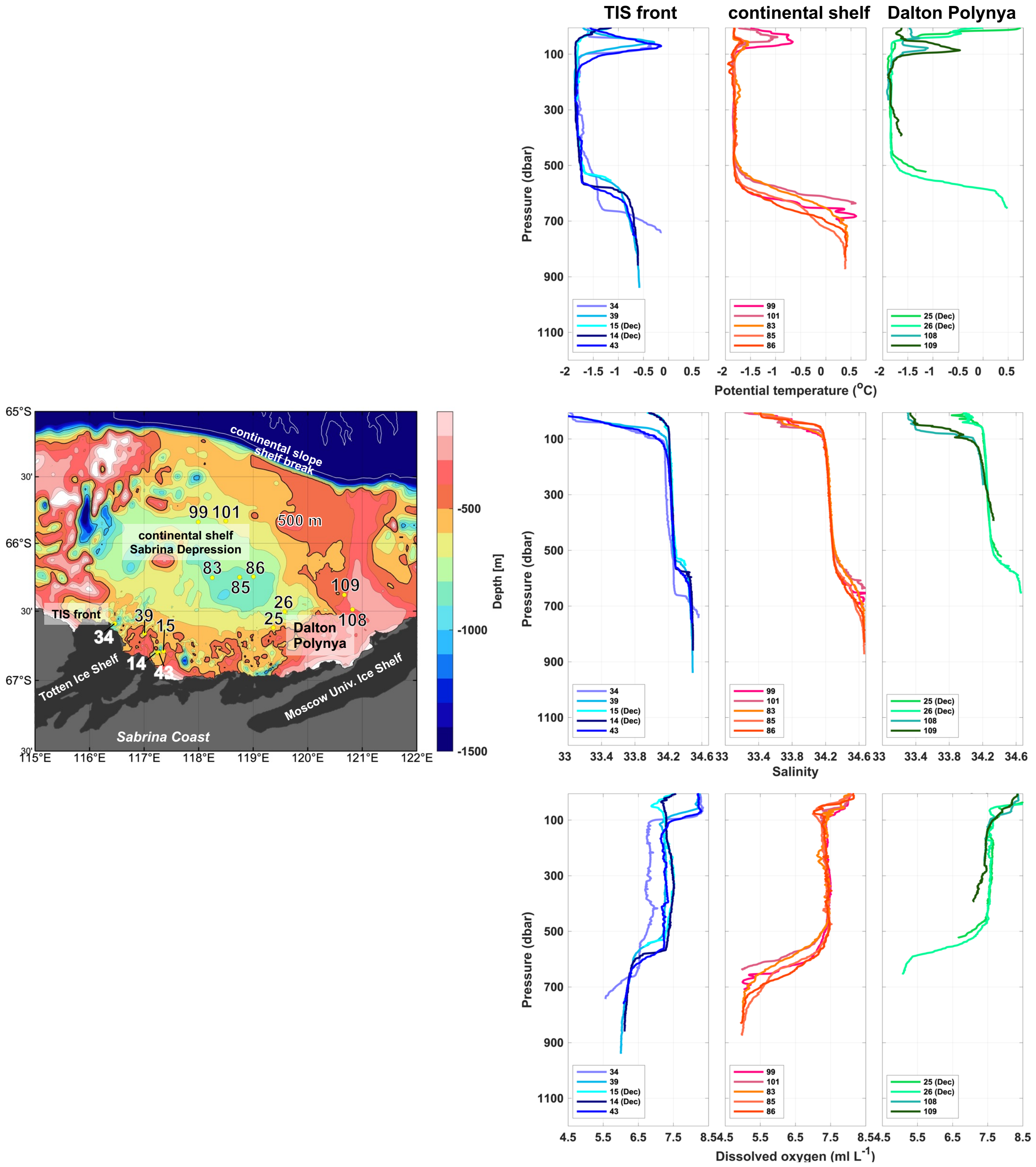

Supplementary Figure 3 | Water mass structure on the Totten continental shelf. Vertical profiles of (upper) potential temperature (°C), (middle) salinity, and (lower) dissolved oxygen (ml L^{-1}) from CTD observations in (left) TIS front (stations 34, 39, 15, 14, and 43), (center) continental shelf (stations 99, 101, 83, 85, and 86), and (right) Dalton Polynya (stations 25, 26, 108, and 109) regions off the Sabrina Coast in December 2019 and February/March 2020 during JARE61. Positions of CTD observations are shown on the map in the left column.

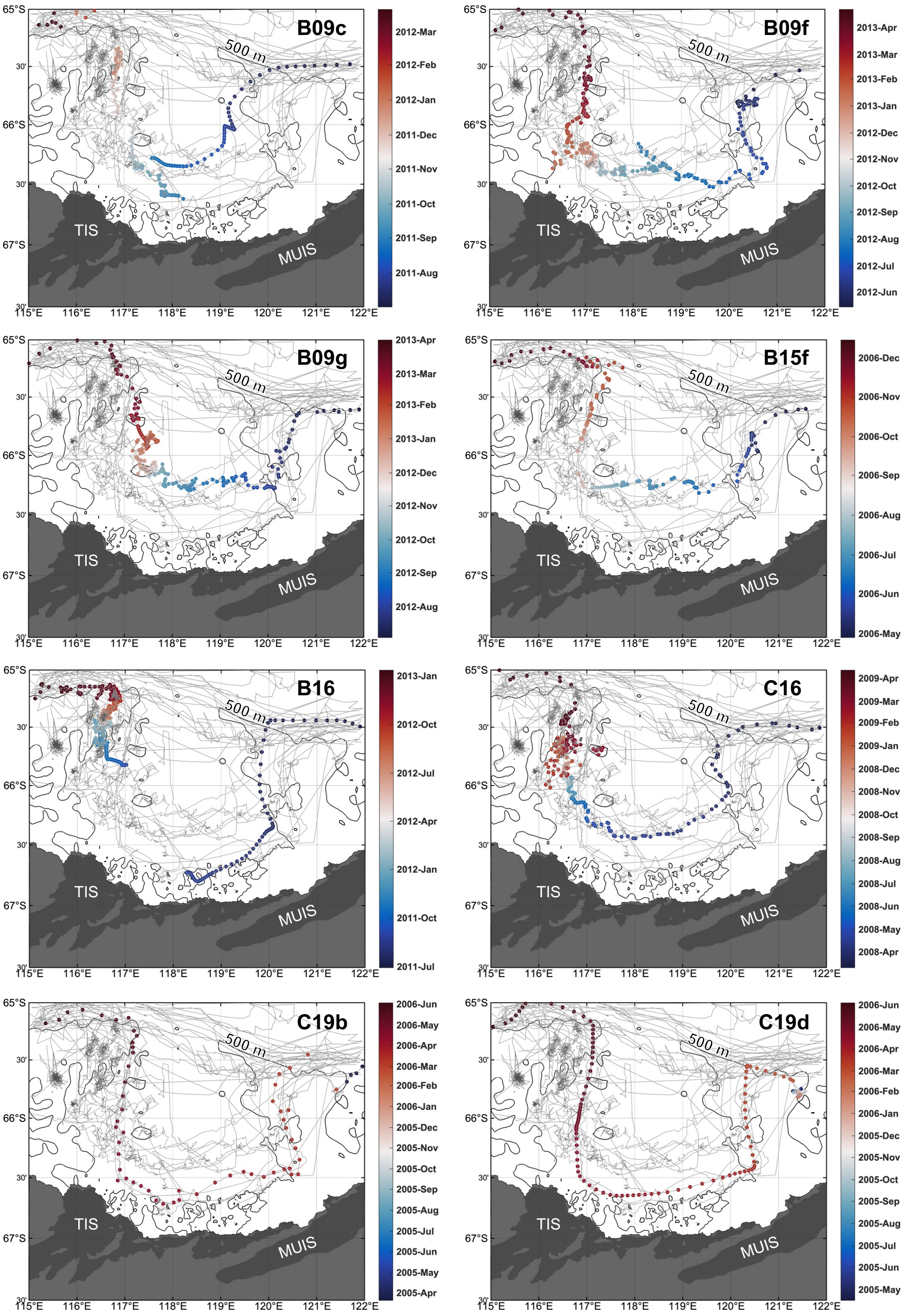

Supplementary Figure 4 | Examples of iceberg drift tracks off the Sabrina Coast. Black lines indicate the available iceberg tracks from the Antarctic Iceberg Tracking Database, Brigham Young University[2]. The colors of symbols represent the passage of time, going from cooler to warmer ones. Thick black line denotes the 500-m isobaths. (TIS: Totten Ice Shelf; MUIS: Moscow University Ice Shelf)

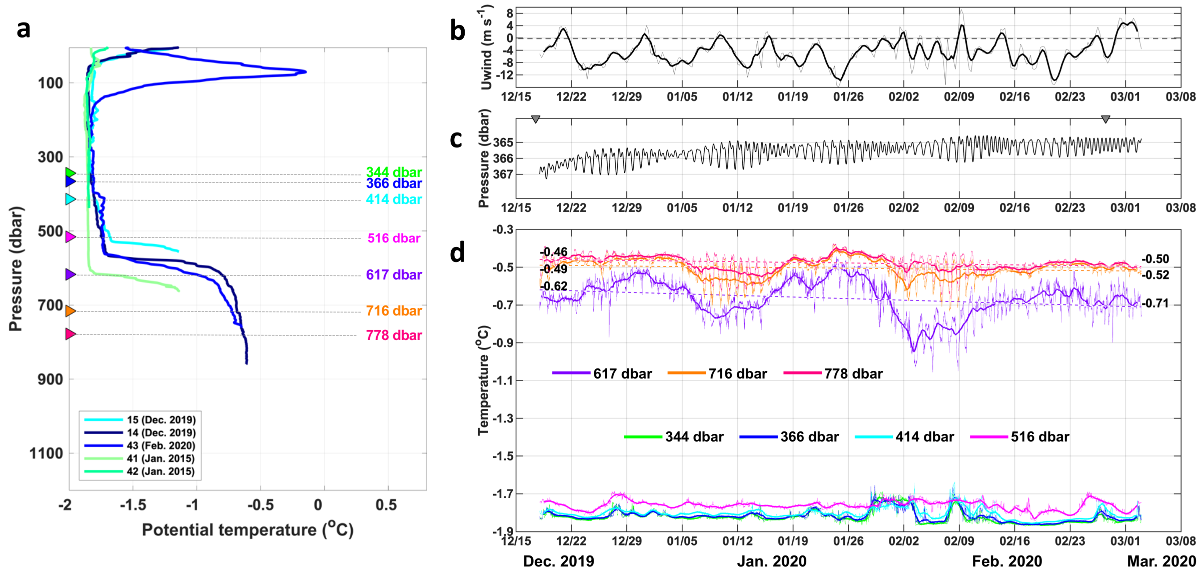

Supplementary Figure 5 | Repeat hydrographic profiles and summertime mooring record in the East Totten Trough. (a) Comparisons of repeat CTD temperature profiles (°C) obtained at East Totten Trough (E-TT) in January 2015 (stations 41 and 42, ANARE)[3] and December 2019/February 2020 (stations 14, 15, and 43, JARE61). See the positions of CTD stations in Fig. 3b. Right-pointing triangles indicate the depths of the moored temperature time series plotted in panel (d), using the same colors (showing which depths the sensors measured). (b) Time series of 6-hourly zonal wind from ERA5 reanalysis (m s^{-1}) averaged over the coastal area between 116 to 121°E and 66.5 to 65.5°S. The bold line indicates smoothed wind record after applying a 25-*h* running average. Time series of (c) water pressure (dbar) and (d) *in-situ* water temperatures (°C) from ~3-month mooring observations from 18 December 2019 to 3 March 2020 at E-TT (almost the same location as station 14, JARE61, see the mooring location in Fig. 3b). The bold lines represent smoothed temperature records after applying a 25-*h* running average. Dashed lines represent the temperature trends over the mooring period (start and end values of the trends are also displayed). Colors correspond to different depths as indicated by the right-pointing triangles in panel (a).

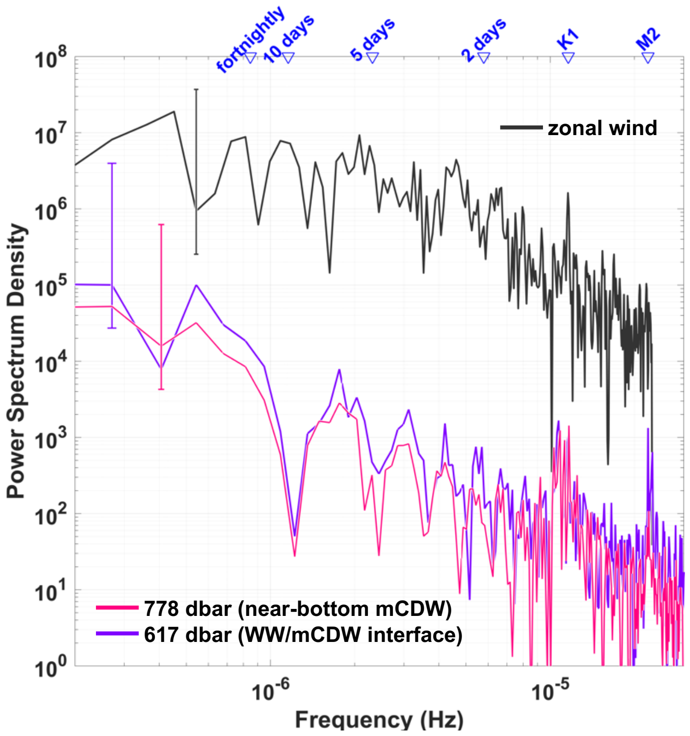

Supplementary Figure 6 | Sub-seasonal fluctuation characteristics of coastal wind and water temperature. Power spectrum density of the zonal wind from ERA5 reanalysis averaged over the coastal area covering from 116 to 121°E and 66.5 to 65.5°S (black) and water temperatures at WW/mCDW interface (purple) and near-bottom mCDW layer (pink). The corresponding time series data are shown in Supplementary Fig. 5. The error bars represent the 95% confidence level.

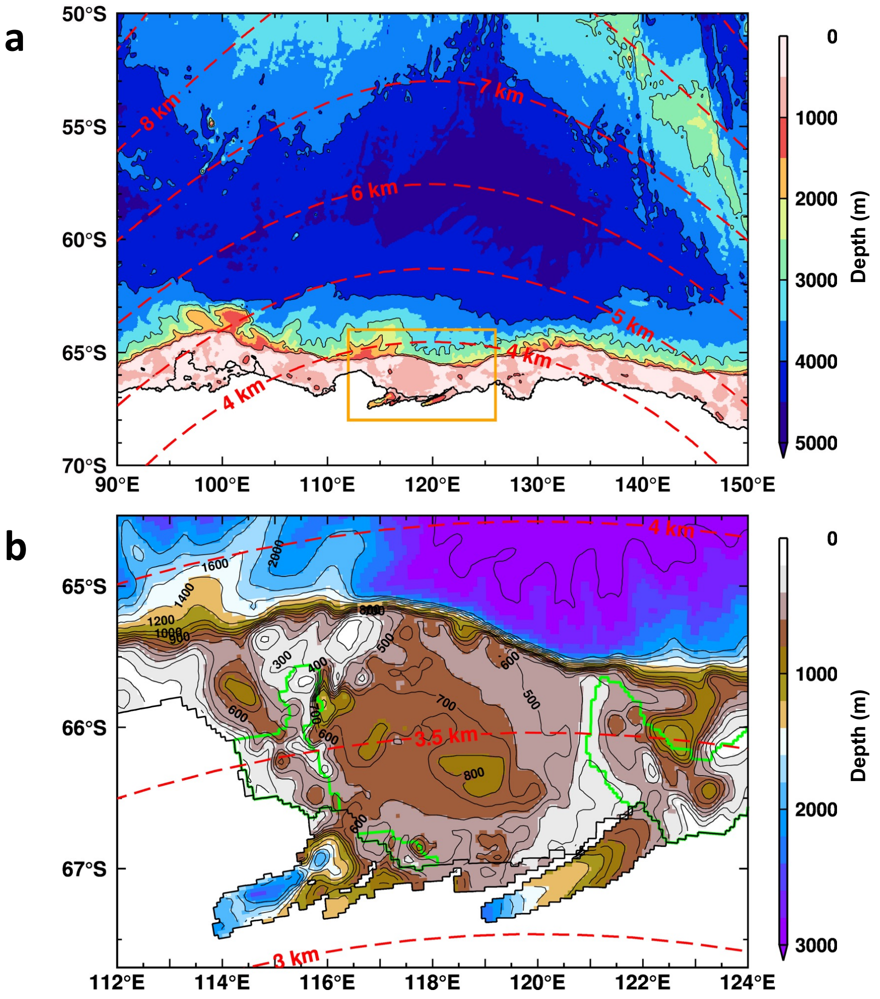

Supplementary Figure 7 | Model's bathymetry and resolution. Model bottom topography (color) and horizontal resolution (red contours) for (a) East Antarctica and (b) off the Sabrina Coast. Green lines in panel (b) show edges of landfast ice[4].

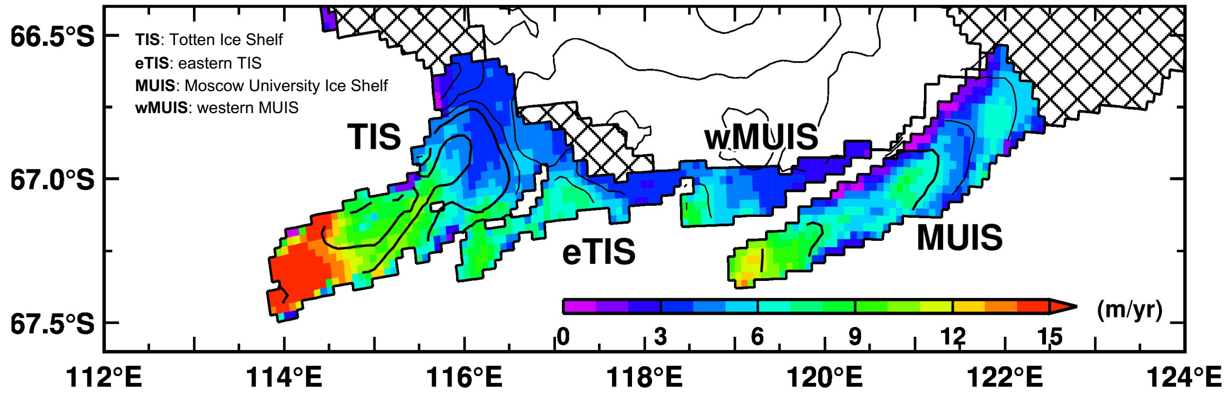

Supplementary Figure 8 | Simulated basal melting at Sabrina Coast's ice shelves. Map of annual-mean ice-shelf basal melt rate (m yr^{-1}) averaged over the period of 1991–2020. Landfast ice grids are masked out with hatch.

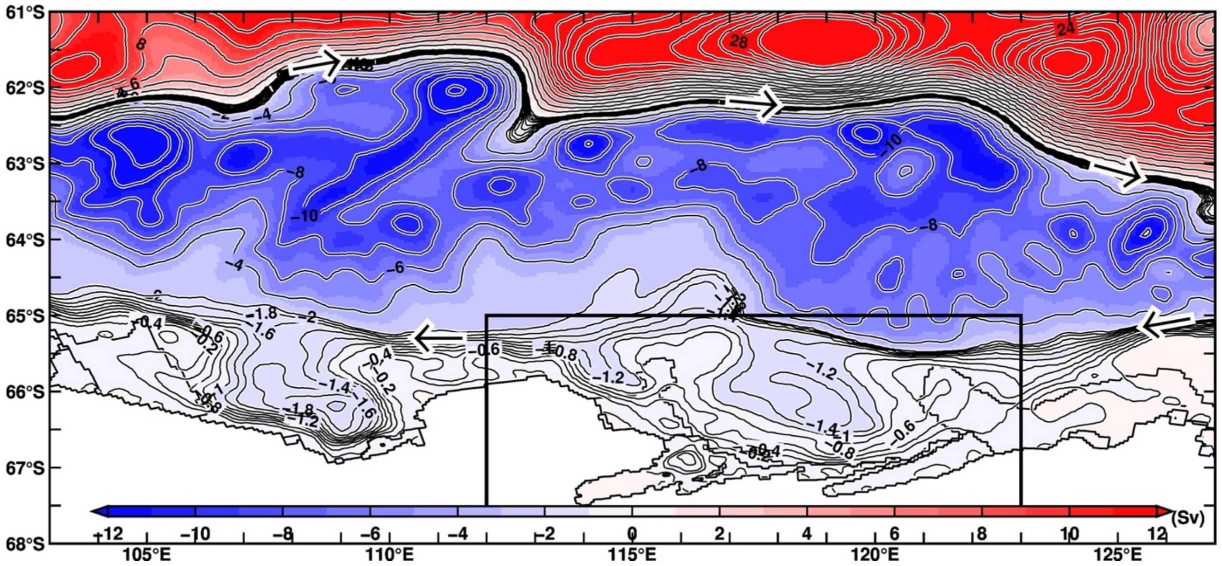

Supplementary Figure 9 | Simulated offshore-to-coast ocean circulation. Streamfunction of vertically integrated transport (Sv, 1 Sv = 1.0×10^6 m^3 s^{-1}) in the model. Arrows show flow directions. The black box shows the area for Fig. 6b. The model results are the annual-mean climatology averaged for 1991–2020.

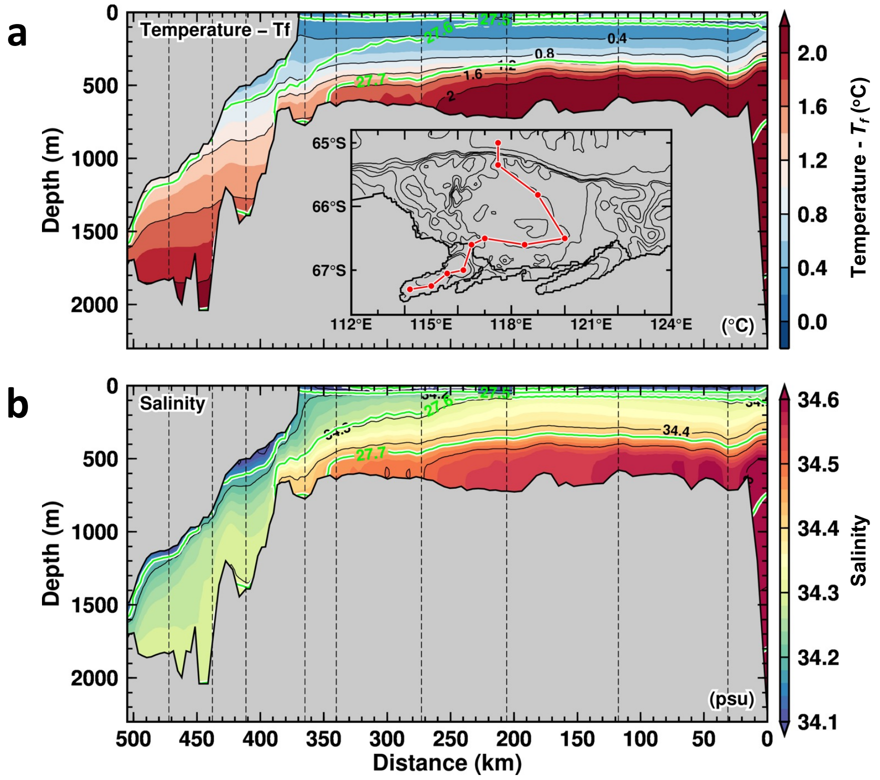

Supplementary Figure 10 | Simulated ocean properties along the warm water pathway. Vertical sections of (a) ocean temperature relative to *in-situ* freezing points (°C) and (b) salinity along the section from the shelf break to the inside of the TIS cavity (inset in panel a showing the defined section).

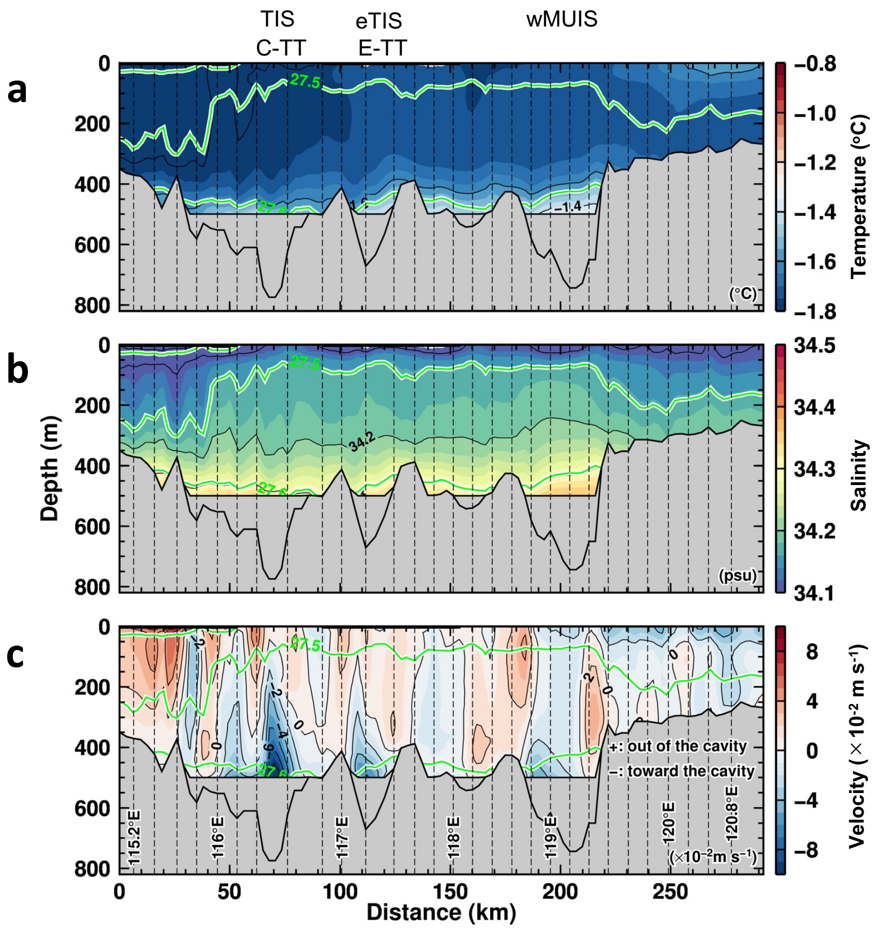

Supplementary Figure 11 | Simulated warm water inflow/meltwater outflow into/out of the Sabrina Coast ice-shelf cavities. As for Figs. 7a–c, but for NOTR case.

Supplementary References:

1. Mizobata K, Shimada K, Aoki S, Kitade Y. The Cyclonic Eddy Train in the Indian Ocean Sector of the Southern Ocean as Revealed by Satellite Radar Altimeters and In Situ Measurements. *Journal of Geophysical Research: Oceans* **125**, e2019JC015994 (2020).

2. Budge JS, Long DG. A Comprehensive Database for Antarctic Iceberg Tracking Using Scatterometer Data. *IEEE Journal of Selected Topics in Applied Earth Observations and Remote Sensing* **11**, 434-442 (2018).

3. Rintoul SR*, et al.* Ocean heat drives rapid basal melt of the Totten Ice Shelf. *Sci Adv* **2**, e1601610 (2016).

4. Fraser AD, Massom RA, Michael KJ, Galton-Fenzi BK, Lieser JL. East Antarctic Landfast Sea Ice Distribution and Variability, 2000-08. *J Climate* **25**, 1137-1156 (2012).
